# Consumer attitudes toward bacteriophage applications to pet food

**DOI:** 10.3389/fvets.2022.921508

**Published:** 2022-08-11

**Authors:** Bailey H. Eagan, Siyun Wang, Nathaniel Hall, Alexandra Protopopova

**Affiliations:** ^1^Animal Welfare Program, Faculty of Land and Food Systems, University of British Columbia, Vancouver, BC, Canada; ^2^Food, Nutrition and Health, The University of British Columbia, Vancouver, BC, Canada; ^3^Department of Food and Animal Sciences, Texas Tech University, Lubbock, TX, United States

**Keywords:** bacteriophage, sustainability, pet food, food safety, environment

## Abstract

This study used a two-part questionnaire to investigate consumer knowledge and attitudes toward bacteriophage applications in pet food, pet food safety, and environmental sustainability. Part 1 included questions about pet food safety, sustainability, and knowledge and attitudes toward bacteriophages. Next, participants reviewed educational materials about each, and Part 2 assessed if this increased knowledge of, or changed attitudes toward, bacteriophage application. Participants (*n* = 80), were recruited through Amazon Mechanical Turk (MT) (*n* = 45) and Social Media (SM) (*n* = 35). Mean responses in Part 1 and Part 2 were compared by paired *t*-tests, and mean responses between MT and SM were compared by *t*-tests. Participants reported pet food safety was important to them (combined proportion strongly agree or agree, mean ± SD) (75/80, 94%, MT 4.66 ± 0.60, SM 4.71 ± 0.95) and were most concerned with raw pet food safety (51/80, 64%, MT 3.88 ± 0.80, SM 3.17 ± 1.40). Participants rated environmental sustainability as important (61/80, 76%, MT 3.86 ± 0.94, SM 3.97 ± 0.66); however, it was not a strong driver of pet food purchasing (26/80, 33%, MT 3.31 ± 1.25, SM 2.82 ± 0.82). Overall, data showed an increase in knowledge of bacteriophages following a review of educational material. However, in the SM group, no statistically significant difference was observed in the comfort eating food with bacteriophage additives (SM Part 1 3.37 ± 1.05, SM Part 2 3.48 ± 1.12, *p* = 0.279), whereas the MT group did show an increase (MT Part 1 3.57 ± 1.01, MT Part 2 4.08 ± 0.92, *p* < 0.001). In the SM group, no statistically significant difference was observed in comfort feeding their pet food with bacteriophage additives (SM Part 1 3.40 ± 1.03, SM Part 2 3.45 ± 1.14, *p* = 0.571), whereas the MT group did show an increase (MT Part 1 3.57 ± 0.98, MT Part 2 4.31 ± 0.84, *p* < 0.001). The strongest objections related to safety concerns (20/53, 38%, MT 2.83 ± 0.96, SM 3.27 ± 0.84). These results demonstrate that despite increasing knowledge, there is still hesitancy among some consumers toward bacteriophage applications in pet food.

## Introduction

Globally, ~50% of households include a companion animal, and pet ownership and related expenditures are continually rising (1, 2). Pet owners often engage in close interactions with their companion including sharing food preparation areas (3, 4). Pathogen contamination in pet food and treats is, unfortunately, possible (5–8). However, Thomas and Feng (3) found that many pet owners in the United States of America (USA) are not aware of risks associated with pet food nor about recalls due to pathogen contamination. The most common bacterial source of foodborne infection worldwide is *Salmonella* (9), and there are documented events of *Salmonella* contamination through household transmission (10), from pet food to pets (11–14), and directly to humans through handling dry pet food and treats (15–17). Furthermore, bacterial pathogen transmission between companion animals and people or through household environments has also been observed in foodborne pathogenic *Staphylococcus aureus* (7, 18–20), *Enterococcus faecium* (21)*, Campylobacter* spp (22, 23), and enteropathogenic *E. coli* (24) [for a comprehensive review see Lambertini et al. (4)].

### Consumer pet food safety practices

Given that contamination is possible, engaging in pet food safety practices is important for maintaining the health and safety of people and their companion animals (16, 25). The Centre for Disease Control (CDC) recommends washing hands with soap and water after handling pet food, storing pet food away from human food, using a clean dedicated scoop for feeding companion animals, and avoiding raw food due to high pathogen prevalence (25). Luckily, compared to non-pet owners in the USA, pet owners have been shown to have better awareness of foodborne pathogens, better handwashing practices, and are more likely to own and use a food thermometer when cooking meat and egg dishes than non-pet owners (26). Compared to dog-only owners, cat owners and cat-dog owners have demonstrated better food safety practices and better handwashing behaviors (26). Despite this, there remain opportunities for improvement in pet food safety among pet owners, and evidence supports a continued need for education about safe pet food handling practices (3, 26, 27); fewer than 25% of pet owners consider dry food a potential threat, and only 58% of owners report washing their hands after feeding their pets (3).

### Consumer attitudes toward environmental sustainability of pet food

Food production, including pet food production, significantly negatively impacts the environment through land use and carbon emissions (28). Pet food production is the main contributor to environmental impacts associated with pet ownership (29). Environmental impacts of pet food are predominately driven by protein sources such as beef and poultry, tin and steel production, and transport (29, 30). In the United States, an estimated 25–30% of the environmental impacts of animal production (including the use of land, water, fossil fuel, phosphate and biocides) are from the production of dog and cat food. Regional estimates such as this assign equal environmental impact to all animal-derived protein sources, including animal by-products (28, 31), therefore may include an over-estimation of environmental impacts (31). However, a global estimate by Alexander et al. (32) of the total environmental impact of dry pet food, including the effects of animal by-products, found that the mean annual greenhouse gas emissions from pet food worldwide was 106 Mt CO_2_ eq—which if this were a country would be equivalent to the 54th highest emitter of greenhouse gas emissions worldwide (33).

The ecological impact of pet food is further increased by modern trends in commercial pet food toward human-grade ingredients competing with the human food supply, and high-protein and nutrient contents, often in excess of nutritional needs (34). While consumers report that the environmental sustainability of pet food is important (35), other factors influence pet food diet choices, such as cost, ingredients, nutritional completeness and recommendation of a diet by a veterinarian (36, 37). Pet food contamination also may reduce the sustainability of the pet food industry due to recalls. Mass production of commercial pet food can result in large batches of contaminated food that lead to recalls and further contributes to global food waste (38). In the last decade, pathogen contamination has resulted in over 221 recalls of pet food and treats (39), such as an outbreak of a multi-drug resistant *Salmonella* infections in dog treats with 154 reported cases in North America (40). Identifying novel methods to reduce pathogen contamination of pet food may benefit human and animal health and reduce the ecological impacts of the pet food industry. However, the effects of food waste due to recalls are likely minor relative to the primary drivers of the environmental impact of pet food production.

### Consumer attitudes toward bacteriophage applications to pet food

Bacteriophages are a type of virus that infects and kills bacteria, are naturally occurring, and are considered one of the most abundant free-living entities on earth. Bacteriophage use has been approved for human consumption by Health Canada and the Food and Drug Administration in the USA (41) as an antimicrobial food processing aid to reduce pathogens in human foods (42). Bacteriophage application may, likewise, help decrease pathogen load in pet food. A bacteriophage preparation (*Salmonella*-specific phage preparation SalmoLyse^®^) has been shown to decrease *Salmonella* in raw pet food by up to 90% (43). Further, a bacteriophage cocktail added to dry pet food kibble has also been shown to decrease the prevalence and concentration of *Salmonella* following the treatment (44).

Despite approval for human consumption, limited research exists about public perception of bacteriophage applications to food, in general. Regarding human consumption of bacteriophages, Cooper (45) noted public concern for adding live viruses to food and suggests a need for more in-depth *in vivo* studies. However, some consumers in the USA reported a willingness to pay for bacteriophage-treated fresh produce for improving food safety indicating a lack of public concern; concern varied by income, race, and state of residence (46). To date, no research has assessed consumer comfort with bacteriophage-treated pet food.

Soffer et al. (43) tested the safety of feeding bacteriophage-treated dry pet food to cats (*n* = 12) and dogs (*n* = 12) by measuring body weight and body condition score, fecal score, food intake, and signs of gastrointestinal issues during 14–15 days of eating bacteriophage-treated dry food. They found no noticeable signs of adverse health effects for dogs or cats, and 93.7% of cats 84% of dogs received “ideal” fecal scores, indicating firm and well-formed feces. Fecal scores rated as “not ideal” were occasionally noted for cats and dogs, primarily indicating soft stools.

Even if current data suggests that bacteriophage application is safe for consumption, there is currently no data to assess consumer attitudes toward bacteriophage applications to pet food. We evaluated consumer knowledge and attitudes toward pet food safety, importance of environmental sustainability of pet food, and pet food bacteriophage applications using a questionnaire approach. Recruitment was conducted from Amazon Mechanical Turk (MTurk, hereinafter “MT”) possibly representing a general lay pet-owning participants, as well as through animal welfare research social media (SM) accounts, likely representing pet-motivated participants. We presented all survey participants with information about bacteriophage use for pet food safety and environmental sustainability and assessed if this information influenced attitudes toward bacteriophage applications. We hypothesized that pet food owners would initially be unaware of bacteriophage applications and that knowledge and comfort with bacteriophages would increase after reading and viewing educational materials.

## Materials and methods

### Questionnaire design

The English-language survey was entered into Qualtrics^XM^ (21), and participants (*n* = 100) were initially recruited in June 2020 using MT (47, 48). Following a modified study design used by Conway and Saker (36) investigating consumer attitudes toward environmental sustainability of grain-free pet food, 78 participants were provided with an initial questionnaire (Questionnaire Part 1 in Supplementary material), completed an educational component including reading an information sheet and watching an informational video on bacteriophages (Information Sheet in Supplementary material), followed by completion of a second questionnaire (Questionnaire Part 2 in Supplementary material). In Part 1 of the survey, participants filled out basic demographic information, including gender, age, level of education, if they were in a life sciences field, if they had a dog and/or cat, and what they fed them for food and treats. Then, participants completed questions about pet food safety, food handling practices, environmental sustainability, knowledge of, and attitudes toward, bacteriophages. The information sheet included information about food safety, environmental sustainability, and bacteriophages. Further, participants reviewed CDC guidance on pet food safety (25) and viewed a video by the National Institute of Allergy and Infectious Diseases (NIAID) on “Fighting Infection With Phages” (49) https://www.niaid.nih.gov/. Finally, participants filled out Part 2 of the questionnaire, which included a repeat of the questions from Part 1 to assess if they learned about bacteriophages from the educational material and if that changed their attitudes toward them. If participants reported having objections to bacteriophage applications, they were provided further questions relating to the specifics of their objections.

Unfortunately, some responses recruited through MT included clear signs of bot activity (50). Answers that were completed improbably quick (defined as under 7 mins) or had unusual, incoherent or duplicate responses in open-ended questions were manually flagged and removed (*n* = 55). As a result, social media recruitment was then conducted in September 2020 through the University of British Columbia's Animal Welfare Program's public Facebook page, resulting in a final combined total of *n* = 80 participants [MT *n* = 45, social media (SM) *n* = 35]. As a result, this provided an opportunity to compare responses between recruitment sources—a general lay pet-owning participants (MT), as well as a pet motivated participant recruited through social media (SM) (a pet owner likely to follow and engage with animal welfare research online). Regardless of recruitment source (MT or SM), all participants completed the same survey through Qualtrics.

Likert-scale questions were used for the survey, and responses were scored numerically as follows; strongly agree (5), agree (4), neither agree nor disagree (3), disagree (2) or strongly disagree (1). Alternatively, questions relating to a frequency included always (5), often (4), sometimes (3), rarely (2) or never (1). Mean response scores were then compared between participants between Part 1 and Part 2 of the questionnaire. Responses were viewed both individually for MT and SM participants and combined.

### Statistical analysis

To compare the 5-point Likert items, paired t-test analyses (51) were conducted to assess if knowledge and consumer attitudes toward bacteriophage applications changed before and after reading the educational materials. Paired t-test analysis was selected based on De Winter et al. (51), showing similar power for analysis of 5-point Likert data between paired t-test and a non-parametric alternative of Mann-Whitney-Wilcoxon analysis. Further, as this study followed a modified study design of Conway and Saker (36) that included paired t-test analysis of 78 respondents before and after reading the educational material, we followed a similar analysis procedure. Paired t-tests were conducted both individually for MT and SM participants and combined. Additionally, t-tests were conducted between MT and SM recruitment sources for all questions to assess if responses differed between source. Multiple tests were run for each question before and after viewing the educational material, and between responses for each recruitment source. We, therefore, used a Benjamini-Hochberg procedure to adjust for multiple comparisons (52), and differences were considered statistically significant at *p* < 0.05. All statistical analysis and data visualization were conducted in R Studio (Version 1.4.1106).

## Results

Of the survey Participants (*n* = 80), participants were recruited through MT (*n* = 45) and SM (*n* = 35). Demographics of participants by each recruitment source are summarized in Table 1.

**Table 1:** Survey participant demographics were separated by each recruitment source of MTurk (*n* = 45) or social media (*N* = 35).

	**MTurk (*n* = 45)**	**SM (*n* = 35)**
**Age**
18–22 years	1	1
23–35 years	17	20
36–55 years	18	9
56–79 years	9	5
**Gender**
Male	26	2
Female	19	32
Non-binary	0	1
**Level of education**
High/trade school	9	5
Postgraduate (Masters, PhD)	12	11
University education	24	19
**Work in a field related to life sciences**		
Yes	10	19
No	35	16
**Life science field**
Animal Medical (DVM, VMD, RVT)	5	7
Human Medical (MD, PA, RN, PhD)	3	3
Research	2	6
Other	1	3
No	34	16
**Have a cat or dog**
Cat	29	24
Dog	38	23
**Feed for meals**
Commercial prepared raw	11	9
Home prepared raw	15	9
Canned wet	25	21
Dry kibble	34	17
Freeze-dried or dehydrated	7	7
Leftovers	12	1
**Feed for treats**		
Commercial prepared raw	10	3
Home prepared raw	8	2
Canned wet	9	3
Dry kibble	14	5
Freeze dried or dehydrated	6	17
Leftovers	9	8
Snack product (e.g., 'Pup-Peroni'	22	9
or 'Milk-bone')

**Figure 1:**
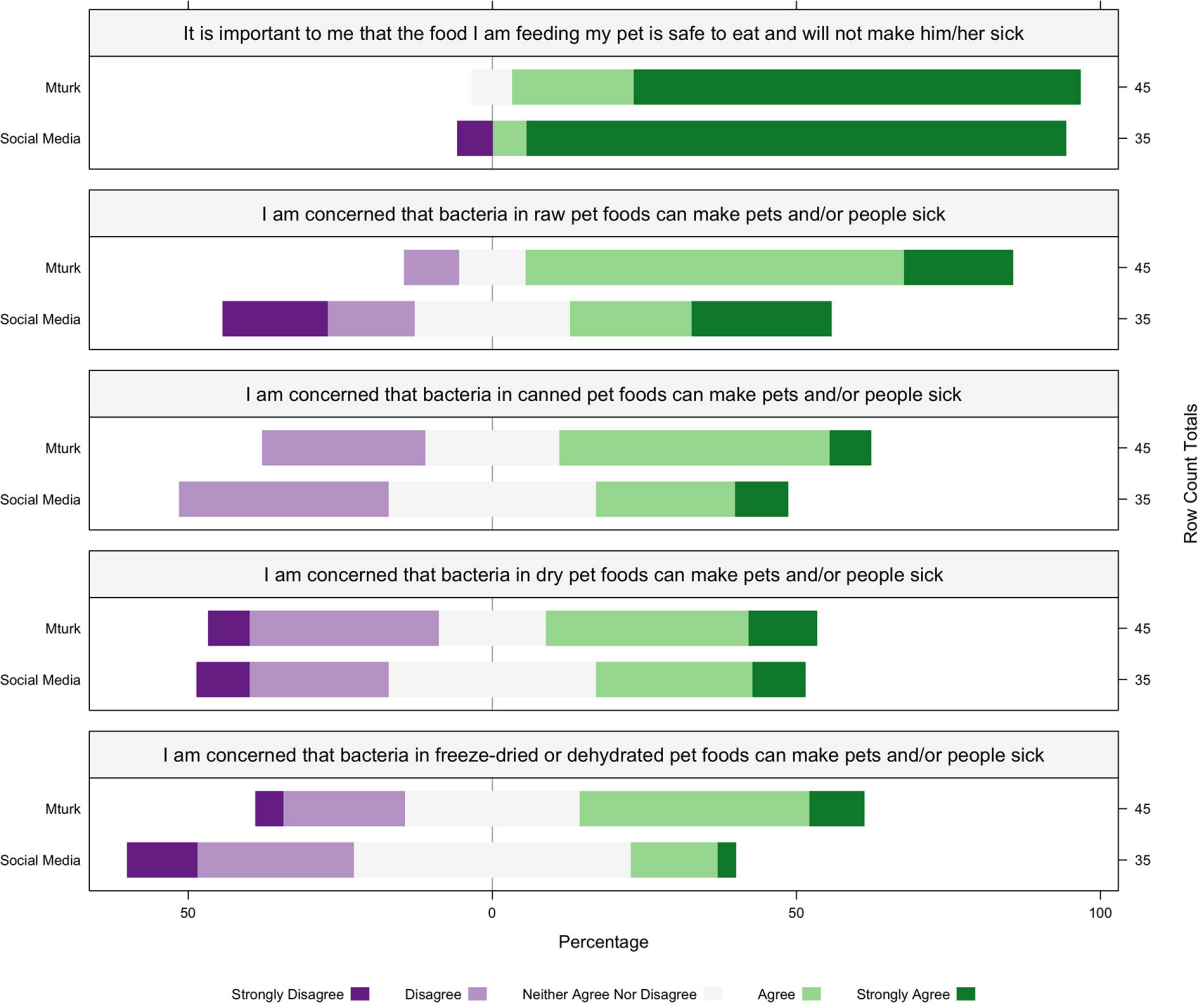
The percentage within each recruitment source of MTurk (MT; *n* = 45) and social media (SM; *n* = 35) of consumer agreement with pet food safety concerns. Participants reported to “Strongly Disagree,”. “Disagree,” “Neither agree Nor Disagree,” “Agree,” or “Strongly Agree” to each pet food safety concern listed in the box above each corresponding plot. The percentage of participants within each recruitment source is represented on the x-axis, the recruitment source is represented on the left y-axis, and the row count totals (the number of participants for each question within each recruitment source) are listed on the right y-axis for each plot.

Overall, 59/80 (74%) of respondents fed dry food [MT 34/45 (75%), SM 25/35 (71%)], 34/80 (43%) fed wet canned food [MT 13/45 (29%), SM 21/35 (60%)] and 30/80 (38%) fed raw food [MT 17/45 (38%), SM 13/35 (37%)] for meals. The majority of participants from both sources reported that they strongly agreed or agreed that it is important the food they are feeding their pet is safe to eat and will not make them sick (combined proportion strongly agree or agree, MT mean ± SD, SM mean ± SD) (75/80, 94%, MT 4.66 ± 0.60, SM 4.71 ± 0.95). Overall, the level of concern that bacteria in raw pet food can make pets and/or people sick was highest (51/80, 64%, MT 3.88 ± 0.80, SM 3.17 ± 1.40), followed by canned (34/80, 43%, MT 3.31 ± 0.94, SM 3.05 ± 0.96), dry (32/80, 40%, MT 3.11 ± 1.17, SM 3.02 ± 1.09), and freeze-dried or dehydrated foods (27/80, 34%, MT 3.26 ± 1.03, SM 2.71 ± 0.95) (Figure 1).

**Figure 2:**
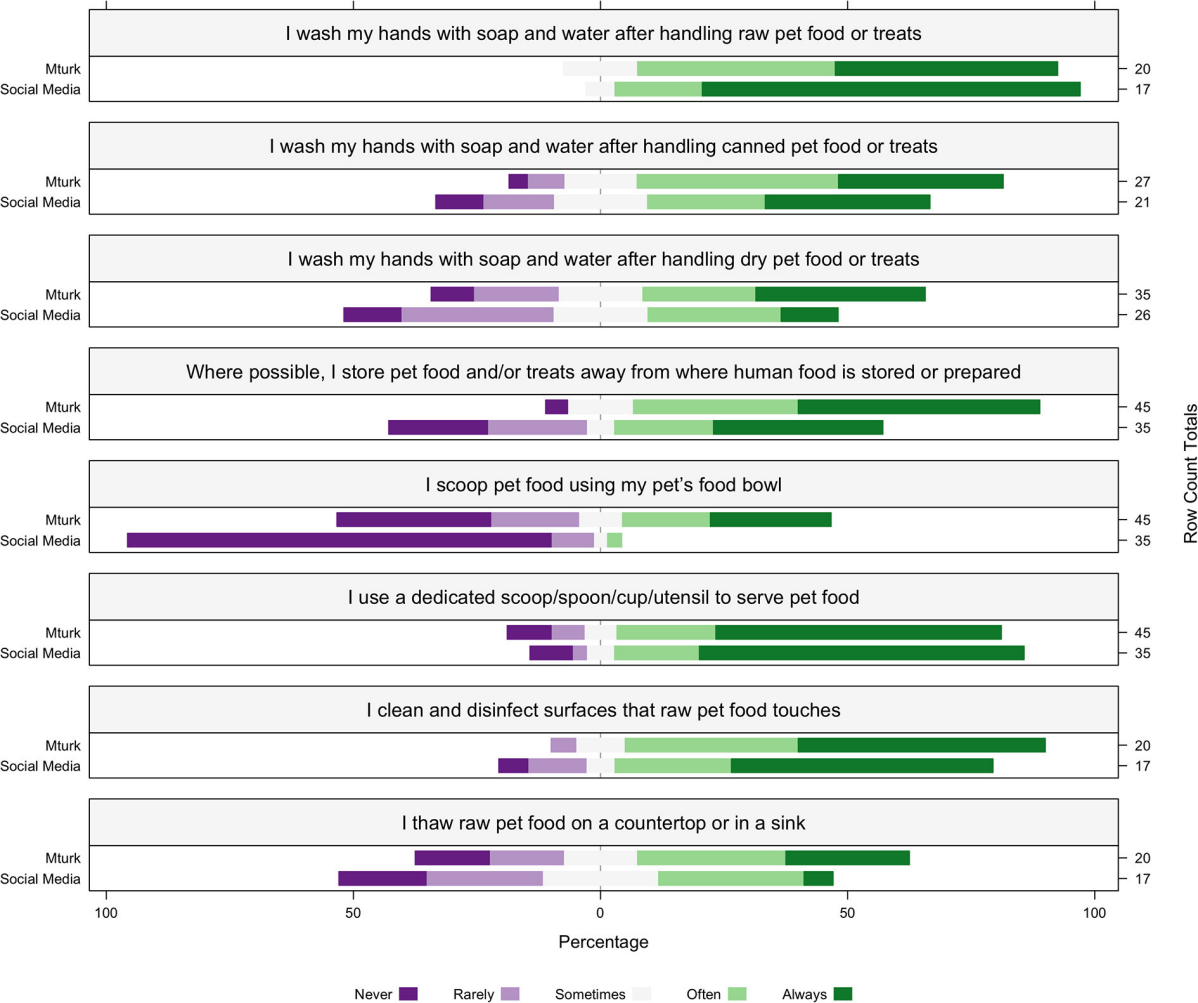
The percentage of participants within each recruitment source of MTurk (MT; *n* = 45) and social media (SM; *n* = 35) that engage in pet food safety practices when feeding pets raw (MT *n* = 20, SM *n* = 17), canned (MT *n* = 27, SM *n* = 21) and dry (MT *n* = 35, SM *n* = 26) pet food and treats. Participants reported to “Never,” “Rarely,” “Sometimes,” “Often,” or “Always” engage in each activity listed in the box above each corresponding plot. The percentage of participants is represented on the x-axis, the recruitment source is represented on the left y-axis, and the row count totals (the number of participants for each question within each recruitment source) are listed on the right y-axis for each plot.

After handling raw pet food, all participants washed their hands with soap and water sometimes or more frequently (combined proportion always, often or sometimes, mean ± SD) (37/37 100%, MT 4.3 ± 0.73, SM 4.7 ± 0.58). Most participants sometimes or often reported washing their hands with soap and water after handling canned pet food (40/48, 83%, MT 3.9 ± 1.07, SM 3.37 ± 1.36) and sometimes when handling dry pet food or treats (41/61, 67%, MT 3.57 ± 1.35, SM 2.96 ± 1.24). Most participants sometimes or often store pet food away from where human food is stored and prepared (64/80, 80%, MT 4.22 ± 0.99, SM 3.28 ± 1.60), never or rarely scoop pet food using a pet food bowl (25/80, 31%, MT 2.86 ± 1.61, SM 1.22 ± 0.64), and often use a dedicated utensil to serve pet food (69/80, 86%, MT 4.11 ± 1.31, SM 4.28 ± 1.25). Of the participants that reported feeding raw food, most participants reported disinfecting surfaces that raw food touches (33/37, 89%, MT 4.3 ± 0.86, SM 4.05 ± 1.29) and sometimes thawing on a countertop or sink (24/37, 64%, MT 3.35 ± 1.42, SM 2.82 ± 1.23) (Figure 2).

**Figure 3:**
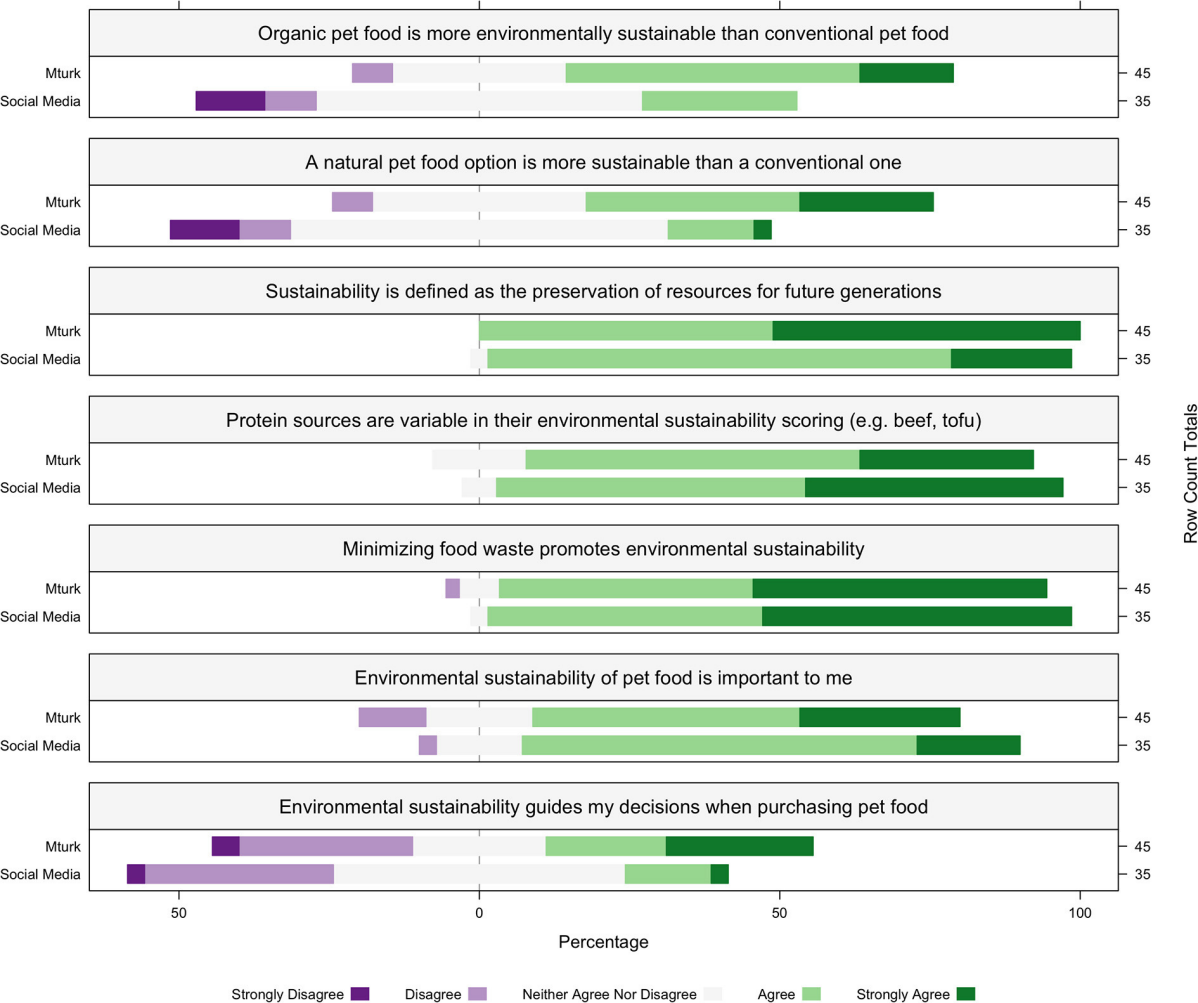
The percentage within each recruitment source MTurk (MT; *n* = 45) and social media (SM; *n* = 35) of consumer agreement with environmental sustainability statements. Participants reported to “Strongly Disagree,” “Disagree,” “Neither agree Nor Disagree,” “Agree,” or “Strongly Agree” to each attitude toward sustainability statements listed in the box above each corresponding plot. The percentage of participants within each recruitment source is represented on the x-axis, the recruitment source is represented on the left y-axis, and the row count totals (the number of participants for each question within each recruitment source) are listed on the right y-axis for each plot.

Regarding the sustainability of pet food, SM participants disagreed more than MT participants whether organic pet food is more environmentally sustainable than conventional pet food (38/80, 48%, MT 3.73 ± 0.80, SM 2.94 ± 0.90) and whether a natural pet food option is more sustainable than a conventional one (32/80, 40%, MT 3.73 ± 0.88, SM 2.88 ± 0.90). Most participants agreed that sustainability is defined as the preservation of resources for future generations (79/80, 99%, MT 4.51 ± 0.50, SM 4.17 ± 0.45). Most participants agreed that protein sources are variable in their environmental sustainability scoring (e.g., beef, tofu) (71/80, 89%, MT 4.13 ± 0.66, SM 4.37 ± 0.59), and that minimizing food waste promotes environmental sustainability (75/80, 94%, MT 4.37 ± 0.71, FB 4.48 ± 0.56). When reporting on the importance of sustainability, most participants agreed that environmental sustainability of pet food is important to them (61/80, 76%, MT 3.86 ± 0.94, SM 3.97 ± 0.66), and disagreed, or neither agreed nor disagreed agreed that environmental sustainability guides their decisions when purchasing pet food (26/80, 33%, MT 3.31 ± 1.25, SM 2.82 ± 0.82) (Figure 3).

Mean responses to bacteriophage-specific questions before (Part 1) and after (Part 2) completing the educational component were compared by paired *t* test. The associated *p* values for each paired t-test following a Benjamini-Hochberg adjustment procedure (52) for combined and separated recruitment sources are included in Table 2.

**Table 2:** Mean responses before and after completion of the educational component of the questionnaire with paired *t*-test statistics and Benjamini-Hochberg (BH) adjusted p values for multiple comparisons.

	**Combined recruitment sources (*****n*** = **80)**			**MTurk (*****n*** = **45)**			**Social media(*****n*** = **35)**		
	**Mean + SD**	**Mean + SD**	**BH**	**Mean + SD**	**Mean + SD**	**BH**	**Mean + SD**	**Mean + SD**	**BH**
	**Part 1**	**Part 2**	**adjusted p**	**Part 1**	**Part 2**	**adjusted p**	**Part 1**	**Part 2**	**adjusted *p***
**Bacteriophage knowledge**									
A bacteriophage is a type of virus that infects and kills bacteria (TRUE)	3.73 + 0.88	4.38 + 0.93	**<0.001**	3.60 + 0.86	4.42 + 0.86	**<0.001**	3.91 + 0.88	4.34 + 1.02	0.041
Bacteriophages are naturally occurring on earth (TRUE)	3.87 + 0.71	4.41 + 0.92	**<0.001**	3.73 + 0.71	4.40 + 0.98	**<0.001**	4.05 + 0.68	4.42 + 0.85	0.041
Bacteriophages are exclusively produced in a laboratory *(FALSE)*	2.45 + 0.97	2.03 + 1.16	**0.001**	2.77 + 1.02	2.24 + 1.33	**0.008**	2.02 + 0.74	1.77 + 0.84	0.087
Bacteriophages are considered one of the most abundant free-living entities on earth (TRUE)	3.46 + 0.74	4.40 + 0.80	**<0.001**	3.71 + 0.66	4.42 + 0.75	**<0.001**	3.14 + 0.73	4.37 + 0.87	**<0.001**
Bacteriophage applications are approved by Health Canada as food processing aids for human consumption (TRUE)	3.21 + 0.63	4.13 + 0.93	**<0.001**	3.42 + 0.62	4.20 + 0.92	**<0.001**	2.94 + 0.53	4.05 + 0.93	**<0.001**
The addition of bacteriophages to foods can decrease bacteria found on the food (e.g., salmonella) (TRUE)	3.47 + 0.81	4.27 + 0.89	**<0.001**	3.64 + 0.77	4.53 + 0.69	**<0.001**	3.25 + 0.81	3.94 + 1.02	**<0.001**
Bacteriophages are highly specific and generally non-toxic to humans, animals and plants (TRUE)	3.52 + 0.81	4.21 + 0.83	**<0.001**	3.66 + 0.82	4.42 + 0.65	**<0.001**	3.34 + 0.76	3.94 + 0.96	**0.001**
Bacteriophage applications to food are odorless and tasteless to humans (TRUE)	3.50 + 0.72	4.16 + 0.89	**<0.001**	3.68 + 0.82	4.37 + 0.71	**<0.001**	3.25 + 0.50	3.88 + 1.02	**0.001**
**Bacteriophage comfort and perception of sustainability**									
I would feel comfortable eating food that had bacteriophage additives	3.48 + 1.03	3.82 + 1.05	**<0.001**	3.57 + 1.01	4.08 + 0.92	**<0.001**	3.37 + 1.05	3.48 + 1.12	0.279
I would feel comfortable feeding my pet food that had bacteriophage additives	3.50 + 1.00	3.93 + 1.07	**<0.001**	3.57 + 0.98	4.31 + 0.84	**<0.001**	3.40 + 1.03	3.45 + 1.14	0.571
I believe bacteriophage additives to food would help environmental sustainability (due to decreased food waste as a result of recalls)	3.52 + 0.95	4.03 + 0.92	**<0.001**	3.68 + 0.87	4.28 + 0.72	**<0.001**	3.31 + 1.02	3.71 + 1.04	**0.001**

**Figure 4:**
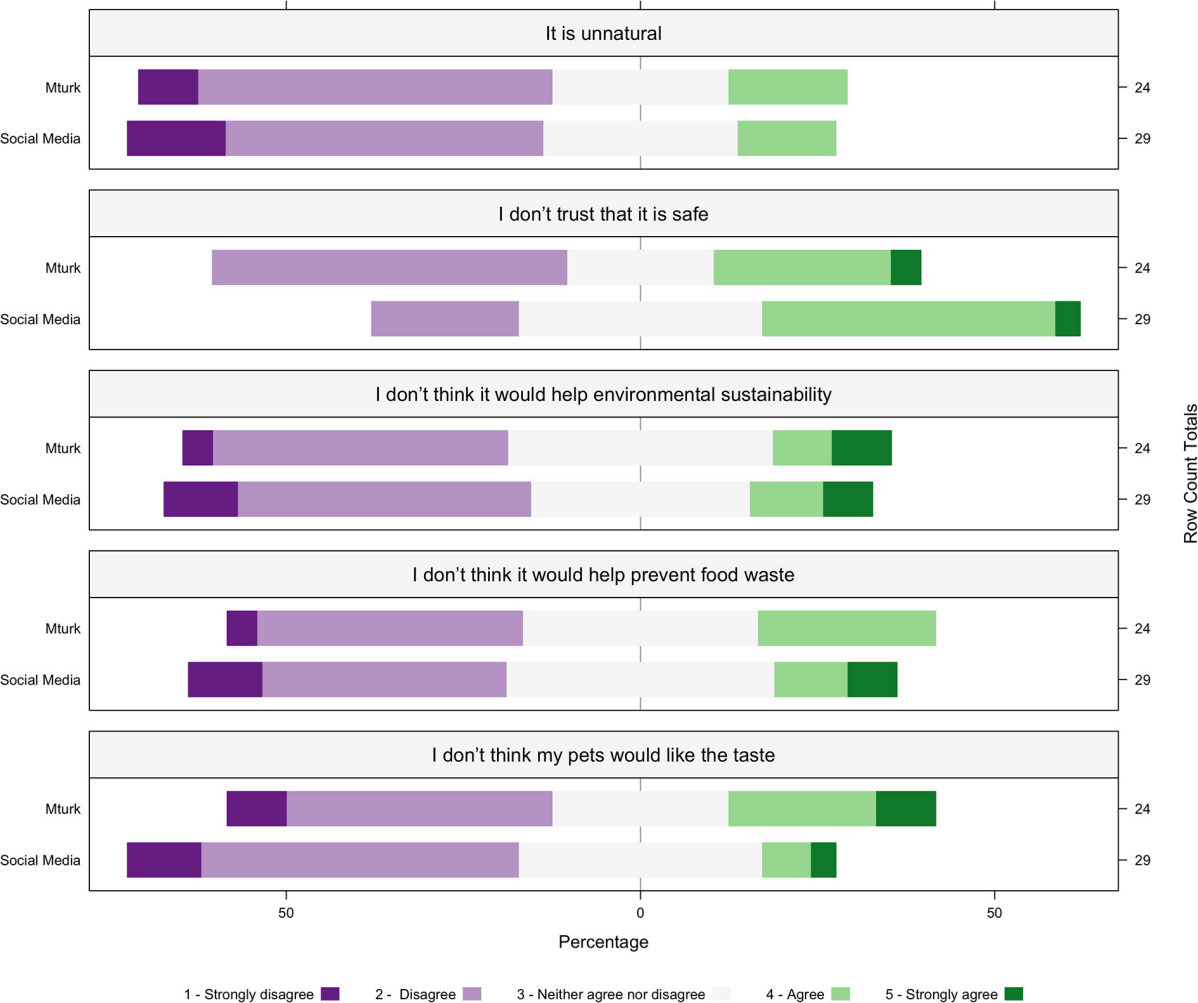
The percentage within each recruitment source MTurk (MT; *n* = 24) and social media (SM; *n* = 29) of consumer agreement with objections to bacteriophage applications. Participants reported to “Strongly Disagree,” “Disagree,” “Neither agree Nor Disagree,” “Agree,” or “Strongly Agree” to each attitude toward sustainability statements listed in the box above each corresponding plot. The percentage of participants within each recruitment source is represented on the x-axis, the recruitment source is represented on the left y-axis, and the row count totals (the number of participants for each question within each recruitment source) are listed on the right y-axis for each plot.

Of participants that reported objections to bacteriophage applications (MT n = 24, SM *n* = 29), the primary objection was they did not trust that it was safe (20/53, 38%, MT 2.83 ± 0.96, SM 3.27 ± 0.84). Comparatively, lower levels of agreement were reported with other objections that participants did not think it would help prevent food waste (11/53, 20%, MT 2.79 ± 0.88, SM 2.68 ± 1.03), did not think their pets would like the taste (10/53, 19%, MT 2.83 ± 1.12, SM 2.48 ± 0.91), did not think it would help environmental sustainability (9/53, 17%, MT 2.75 ± 0.98, SM 2.62 ± 1.04), or believed that bacteriophage applications are unnatural (8/53, 15%, MT 2.5 ± 0.88, SM 2.41 ± 0.90) (Figure 4).

Of the 35 participants (MT = 14, SM = 21) who provided additional information to open-ended question, 24 (MT = 10, SM = 14) cited safety concerns or a need for more technical information. Further, 6 participants in the SM group directly indicated that the study by Soffer et al. (43) conducted on a sample size of 12 cats and dogs is not enough data to feel comfortable with bacteriophage applications.

Statistically significant differences in responses between recruitment sources MT and SM are summarized in Table 3.

**Table 3:** Statistically significant (*p* < 0.05) differences between mean responses of recruitment sources MTurk (MT) and social media (SM) responses following adjustment using the Benjamini-Hochberg procedure.

	**Combined recruitment sources (*****n*** = **80)**		
	**MT**	**SM**	**BH adjusted *p***
	**Mean + SD (*n* = 45)**	**Mean + SD (*n* = 35)**	
**Pet food safety concerns and practices**			
I am concerned that bacteria in raw pet foods can make pets and/or people sick	3.88 + 0.80	3.17 + 1.40	**0.033**
I am concerned that bacteria in freeze dried or dehydrated food and treats can make pets and/or people sick	3.26 + 1.03	2.71 + 0.95	**0.045**
Where possible, I store pet food and/or treats away from where human food is stored or prepared	4.22 + 0.99	3.28 + 1.60	**0.021**
I scoop pet food using my pet's food bowl	2.86 + 1.61	1.22 + 0.64	**<0.001**
**Environmental sustainability**			
Organic pet food is more environmentally sustainable than conventional pet food	3.73 + 0.80	2.94 + 0.90	**0.002**
A natural pet food option is more sustainable than a conventional one	3.73 + 0.88	2.88 + 0.90	**0.001**
Sustainability is defined as the preservation of resources for future generations	4.51 + 0.88	4.17 + 0.90	**0.015**
**Bacteriophage questions part 1**			
Bacteriophages are exclusively produced in a laboratory	2.77 + 1.02	2.02 + 0.74	**0.003**
Bacteriophages are considered one of the most abundant free-living entities on earth	3.71 + 0.66	3.14 + 0.73	**0.004**
Bacteriophage applications are approved by Health Canada as food processing aids for human consumption	3.42 + 0.62	2.94 + 0.53	**0.003**
Bacteriophage applications to food are odorless and tasteless to humans	3.68 + 0.82	3.25 + 0.50	**0.023**
**Bacteriophage questions part 2**			
Addition of bacteriophages to foods can decrease bacteria found on the food (e.g., salmonella)	4.53 + 0.69	3.94 + 1.02	**0.020**
Bacteriophages are highly specific, and generally non-toxic to humans, animals and plants	4.42 + 0.65	3.94 + 0.96	**0.045**
Bacteriophage applications to food are odorless and tasteless to humans	4.37 + 0.71	3.88 + 1.02	**0.041**
I would feel comfortable feeding my pet food that had bacteriophage additives	4.31 + 0.84	3.45 + 1.14	**0.029**
I believe bacteriophage additives to food would help environmental sustainability (due to decreased food waste as a result of recalls)	4.28 + 0.72	3.71 + 1.04	**0.029**

*Bold BH indicates that the p designates a statistically significant difference between Part 1 and Part 2 following a Benjamini-Hochberg (BH) adjustment for multiple comparisons (p <0.05)*.

## Discussion

### Consumer attitudes toward bacteriophage applications to pet food

This study aimed to assess pet owner knowledge and attitudes toward bacteriophage applications in pet food, pet food safety, and importance of environmental sustainability of pet food. To assess attitudes toward bacteriophage additives to food, we tested if pet food consumers reported increased comfort in feeding their companion animals bacteriophage applications or eating bacteriophages after learning about them. Prior to reviewing educational materials, most participants reported neutral responses (i.e., “3-neither agree nor disagree”) to the statements regarding eating and feeding bacteriophage additives. After reviewing the educational material, the MT group showed increased knowledge of and comfort with bacteriophage additives. However, despite increased knowledge of bacteriophages following reviewing educational material about them, contrary to the hypothesis that this would increase comfort for all participants, minimal differences were observed in the SM group in comfort eating or comfort feeding bacteriophage-treated food to their companion animals.

This study opportunistically allowed for comparing responses from different recruitment sources: MT and SM. The MT participants may have represented a general lay audience with a greater proportion of dog owners. However, the demographic information shows that the MT group differs from larger sample survey studies (3, 26), therefore the MT group may not be entirely representative of a general pet-owning participant, and this limitation is discussed in further detail below. The SM group presumably represented particularly pet-motivated participants, and included a greater proportion of cat-owning women working in a life science field than the MT group. While overall results showed similar trends between groups, a few distinct differences are discussed in detail below.

As indicated by an overall increase in identifying bacteriophage truisms in Part 2 compared to Part 1 of the questionnaire, overall, combined source participants showed increased knowledge about bacteriophages after viewing the materials, increased comfort eating food with bacteriophage additives, and feeding their pets food that had bacteriophage additives. There is currently no direct evidence demonstrating that bacteriophages may help environmental sustainability of pet food due to decreased recalls, however between Part 1 and Part 2, there was a combined overall increase in the belief that bacteriophage additives would help environmental sustainability due to decreased recalls. Relative to the predominant drivers to the environmental impacts of pet food production including beef and poultry protein, tin and steel production, and transport (29, 30), it is likely that food waste as a result of recalls is a relatively small contributor to overall impacts.

While the MT group demonstrated increased knowledge of bacteriophages and comfort eating and feeding pets bacteriophage additives, the SM group did not demonstrate these same trends. The SM group showed some increase in knowledge about bacteriophage applications between Part 1 and Part 2, however some differences were not statistically significant, apparently due to already high knowledge about bacteriophages in response to some questions. As this group contained a high number of participants working in life science fields, they may have had higher initial knowledge about bacteriophages, however this pattern was not consistent across all responses. Despite increasing knowledge regarding bacteriophages demonstrated in the SM group between Part 1 and Part 2, the SM group did not demonstrate statistically significant increases in comfort eating or feeding their pet food with bacteriophage additives as demonstrated in the MT group.

Statistically significant differences were observed between recruitment source responses to Part 1 and Part 2 of the survey. Most notably, the MT group demonstrated a greater increase in knowledge between Part 1 and Part 2, ultimately showing higher agreement with bacteriophage truisms in Part 2 than the SM group. Further, the MT group reported higher comfort with feeding pet food that had bacteriophage additives than the SM group, and a stronger belief that bacteriophage additives would help environmental sustainability due to decreased recalls.

Among objections, the most common concern among participants was that they do not trust that bacteriophage applications are safe (38%). Relatively fewer participants reported they do not think it would help prevent food waste (20%), do not think pets would like the taste (19%), do not think it would help environmental sustainability (17%), or believe bacteriophages are unnatural (15%). Among the most common objection of not trusting that bacteriophages are safe, SM participants agreed the most with this, however, there was no statistically significant difference between the SM and MT group, and safety concerns were also the primary objection among the MT group. The lack of overall change in comfort eating or feeding pets bacteriophages in the SM group, and the primary objection from both groups after learning about them being safety concerns, may also suggest a need for further safety data or information for consumers.

While bacteriophages are an approved food processing aid in Canada (42), some public concern has been reported regarding adding live viruses to food for consumption (45). The safety of feeding bacteriophages has been tested by Soffer et al. (43). While results showed no noticeable signs of adverse health effects for dogs or cats, the limited amount and extent of research may contribute to hesitancy. It is possible that further bacteriophage research demonstrating safety may not increase comfort, as seen with common drivers of vaccine hesitancy (53), for example. Overall, 24/35 of the participants replied to open-ended questions citing safety concerns, and 6 specifically mentioned the single study by Soffer et al. (43) on 12 cats and dogs not being enough of a body of research to inform their decision. This likely indicates that further studies assessing the safety of feeding bacteriophage additives may be beneficial to increasing comfort. However, while the primary objection was safety, only 38% of respondents reported this concern. Therefore, additional hesitancy drivers that were not assessed in this study likely contribute to concerns about bacteriophages. For example, it is possible some respondents were not convinced enough of the need for bacteriophages in pet food to justify the consideration of their functions. Further research evaluating other objections to bacteriophage applications would be beneficial to inform the prevalence and sources of public concern.

### Consumer pet food safety practices

In addition to assessing attitudes toward bacteriophage applications, we assessed consumer attitudes and practices relating to pet food safety and environmental sustainability of pet food. Consistent with Thomas and Feng (3), most survey participants in the present study (94%) reported pet food safety is important to them. Generally, the majority were unaware that pet food can have pathogens (except raw food, where 64% noted concerns). Knowledge of other food types posing a risk to human and animal health was lower for canned (43%), dry (40%) and freeze-dried or dehydrated (34%) pet food. Thomas and Feng (3) found that <25% of survey participants knew that dry food might contain pathogens. While the reported awareness of pathogens in pet food in the present study was higher (34–64% depending on food type) than observed in Thomas and Feng (3), these results maintain that there are opportunities for food safety education among consumers.

While some participants followed pet food handling practices, others did not. The present findings are in line with other research (3, 26) that suggests room for improvement in pet food handling education. Food-type-specific levels of concern appear to dictate pet food handling practices, as 100% of participants reported washing their hands with soap and water sometimes, often or always after handling raw food. Thomas and Feng (3) found that 58% of owners reported washing their hands after feeding pets, less than reported by participants in the present study (raw 100%, canned 83%, and dry 67%). Additionally, the present findings show that 80% of people store pet food away from human food, 69% do not scoop food using the pet's bowl, 86% use a dedicated utensil. When feeding raw food, 89% disinfect surfaces raw food touches and 64% thaw raw food on a countertop or sink. Generally, most participants are engaging in some pet food safety practices. However, there are opportunities for increased education, especially relating to non-raw foods where safe handling behaviors appear less important to owners.

Overall, the SM participants were less concerned with bacterial contamination of pet foods than MT participants, specifically regarding raw and freeze-dried pet food and treats, and less likely to store pet food and treats away from where human good is stored and prepared. However, the SM group were also less likely to scoop pet food using the pet's bowl. Considering the higher proportion of cat-owners in the SM group, the less concern and lower likelihood to store pet food away from human food contradicts Ma et al. (26) who found cat-owners more likely to engage in food safety practices than dog-owners. The reasons for this difference in the present study are unknown, research investigating motivations for failing to engage in pet food safety practices would be beneficial. Considering the lower reported level of concern in the SM group compared to the MT group, it could be speculated that the increased hesitancy in the SM group to eat or feed bacteriophage applications could be due to this lower level of concern with pathogens in food (therefore perhaps considering bacteriophage applications not necessary). However, this specific objection was not assessed in the present study, and future research should address this possibility.

### Consumer attitudes toward environmental sustainability of pet food

A 2020 industry survey of pet food market trends in the USA showed that 15–28% of people (range between age categories) are interested in seeing more sustainably sourced pet food, while 10–25% are interested in seeing pet food with plant-based protein (54). Conway and Saker (36) found that pet food's environmental sustainability is important to consumers, but other factors including health, cost, ingredients, nutritional completeness and recommendation of a diet by a veterinarian influenced the likelihood to change a pet's diet (55). A similar trend was reflected in the present study. While participants agreed with the given definition of sustainability, that minimizing food waste promotes environmental sustainability, that protein sources are variable in their environmental sustainability, and that sustainability is important to them, sustainability did not firmly appear to guide purchasing decisions in participants from either recruitment source. Further research directly investigating why the environmental sustainability of pet food is important to consumers but does not appear to be a strong motivator in purchasing choices would be informative.

One possible explanation for this discrepancy could be the difficulty of determining the environmental sustainability of a food item due to the many possible metrics (e.g., greenhouse gas emissions, land, water and resource consumption) and further communicating that information to consumers (56). Indeed, obstacles exist in assessing and labeling food for fostering sustainable dietary habits (55). Pet food production has substantial and continually increasing environmental impacts globally (28, 29, 31, 32). Given the scale of the issue, continued efforts, such as those to assess the environmental impacts of pet food (28, 29, 31, 32), are critical. Additionally, using that information to make changes in the pet food production system and communicate this information from pet food suppliers to consumers to inform sustainable purchasing practices is needed. Pet owners may understandably not have clarity on the environmental impacts of pet food and how to purchase pet food sustainability; therefore, other factors may motivate these choices. Further research into what drives pet food purchasing and how sustainable pet food manufacturers can incorporate these drivers must be prioritized.

### Limitations, conclusions, and future directions

This study included some limitations and many opportunities for further research. First, as this survey was distributed only to English-speaking consumers in North America, this does not assess differing perspectives and opinions of non-English speaking pet owners. Additionally, recruitment included participants from two different sources due to a bot activity in the initial MT recruitment. This ultimately presented a unique opportunity to observe apparent differences between groups, a general lay pet-owning participant and a pet motivated participant. However, compared to two large survey studies in the USA conducted by Ma et al. (26) and Thomas and Feng (3) including 2,285 and 1,040 pet owning participants respectively, there is some indication that the MT group in the present study is not representative of the general pet owning population. Specifically, in the present study, 53% of MT participants had a university education, and 26% had a graduate degree, which are higher proportions than those seen in larger-sample studies (3, 26). The MT group shows 11% of participants in the veterinary field, which exceeds likely proportions based on national employment by industry averages (57, 58). As a result, the MT group responses may not be generalizable to a lay pet-owning respondent. Further, overall, 38% of respondents fed their pets raw food for meals, which is higher than 25% reported in Thomas and Feng (3), indicating that pet owners feeding raw food diets may be overrepresented in the present study.

As indicated by an overall increase in knowledge and comfort in Part 2 compared to Part 1, as well as participant confirmation they had reviewed the educational material, it was assumed each participant reviewed all information following Part 1 of the survey. However, it is possible that participants did not review all details in the Educational information section. Survey results also may have been impacted by attribute priming (59), and some participants may have been impacted by a bias toward social desirability of a positive response (60, 61). This may have contributed to, for example, the discrepancy between reported importance of environmental sustainability while reporting that it did not guide purchasing decisions.

While this survey assessed comfort with bacteriophages and presented common expected objections to bacteriophage applications, the primary objection of safety was reported in only 38% of participants. Therefore, additional drivers are likely contributing to bacteriophage hesitancy in the present study that were not assessed, and future research investigating further objections would be beneficial for understanding consumer concern surrounding bacteriophages. Furthermore, while this study collected information on the importance of sustainability and if awareness of sustainability guides pet food purchasing, this study did not assess other possible drivers of pet food purchasing.

Overall, only the MT group showed increased participant comfort with eating bacteriophage-treated food and feeding bacteriophage-treated food to their pets after learning about bacteriophages, while the SM group showed very minimal statistically insignificant increases in comfort. These results suggest that there may be a need for further safety data, specifically among pet-motivated participants likely represented by the SM group. Studies assessing the safety of pet food bacteriophage applications would likely inform consumer comfort. Additionally, opportunities exist to increase knowledge of pet food safety to improve pet food safety practices, especially with canned, dry and freeze-dried foods. The observed lower level of concern about pathogens in pet food in the SM group suggests that pet-food safety educational information explicitly targeted to pet-motivated participants through social media would likely be beneficial in improving pet food safety practices. Finally, while overall participants rate environmental sustainability as important to them, it is not a strong driver of pet food purchasing. As pet ownership continually increases, and worldwide, humans live closely with their pets, bacteriophage applications may play an essential role in minimizing pathogen contamination. However, overall, consumer attitudes suggest a need for further studies demonstrating their safety before they may be readily accepted into pet diets by consumers.

## Data availability statement

The original contributions presented in the study are included in the article/Supplementary material, further inquiries can be directed to the corresponding author.

## Ethics statement

The studies involving human participants were reviewed and approved by University of British Columbia's Behavioural Research Ethics Board (H20-00608). The patients/participants provided their written informed consent to participate in this study.

## Author contributions

BE, SW, NH, and AP contributed to the conception of the study and subsequent study design. BE deployed the survey, compiled results, and wrote the first draft of the manuscript. BE and AP performed the statistical analysis. All authors contributed to the manuscript revisions and approved the final version of the manuscript for submission.

## Funding

The study was funded by the University of British Columbia Faculty of Land and Food Systems Internal Research Grant Program (F20-00710).

## Conflict of interest

The authors declare that the research was conducted in the absence of any commercial or financial relationships that could be construed as a potential conflict of interest.

## Publisher's note

All claims expressed in this article are solely those of the authors and do not necessarily represent those of their affiliated organizations, or those of the publisher, the editors and the reviewers. Any product that may be evaluated in this article, or claim that may be made by its manufacturer, is not guaranteed or endorsed by the publisher.

## Supplementary material

Supplementary Data Sheet 1 Github link for access to R code used to analyze consumer attitudes toward bacteriophage applications for food safety and environmental sustainability of pet food.

Supplementary Data Sheet 2 Bacteriophage Qualtrics survey and information sheet.

Supplementary Table 1 Qualtrics survey data.
