# Supplementary material for: Consumer attitudes toward bacteriophage applications to pet food
Source: Front Vet Sci. 2022 Aug 11;9:921508. doi: 10.3389/fvets.2022.921508 (PMC9403512; doi:10.3389/fvets.2022.921508)
Supplement: Supplementary Data Sheet 1 — Github link for access to R code used to analyze consumer attitudes toward bacteriophage applications for food safety and environmental sustainability of pet food. [file Data_Sheet_1.PDF]

SUPPLEMENTARY DATA SHEET 1

Access to R code for “Consumer Attitudes Toward Bacteriophage Applications for Food Safety and Environmental Sustainability of Pet Food” available at:
https://github.com/baileyhe/bacteriophagesurvey.git
