# Supplementary material for: Consumer attitudes toward bacteriophage applications to pet food
Source: Front Vet Sci. 2022 Aug 11;9:921508. doi: 10.3389/fvets.2022.921508 (PMC9403512; doi:10.3389/fvets.2022.921508)
Supplement: Supplementary Data Sheet 2 — Bacteriophage Qualtrics survey and information sheet. [file Data_Sheet_2.PDF]

Information Sheet:

Food safety, environmental sustainability and the use of bacteriophage applications in pet food

Food Safety

Exposure to pathogens causes gastrointestinal illness in both pets and people [1], and handling contaminated pet food and treats (including raw and dry food) is a known source of infection [2–4]. Following pet food safety practices is important for maintaining health of both people and their pets [4,5].

Please review the Centre for Disease Control (CDC) Pet Food Safety Information Sheet (click here)

Environmental Sustainability

Food production, especially animal food production is a large contributor to climate change. The world's food supply is affected not only by human consumption, but also pet food consumption[6,7]. Worldwide, an estimated 500 million domestic pets in the world consume up to 30% of animal production products[7,8]. While previously pet food was composed of non-human-grade products and waste, the environmental impact of pet food is increased by modern consumer trends toward human-grade ingredients and high-protein and nutrient contents, directly competing with the human food system [6].

Another consideration regarding the environmental sustainability of pet food is food waste. Human food waste has become a growing concern with the recall rate of food going up 10% in the last 5 years [9]. Although no centralized databases exist that allow the quantification of wasted pet food, the amount can be inferred from industry-announced recalls. Due to pathogen contamination, in the last decade, pet food companies have issued at least 221 recalls 10 including an outbreak of a multi-drug resistant Salmonella infections in dog treats with 154 reported cases in North America [11].

Bacteriophages

Bacteriophages (or phages) are a type of virus that infects and kills bacteria. Bacteriophages are naturally occurring, and are considered one of most abundant free-living entities on earth [12].

Please watch the following video by the National Institute of Allergy and Infectious Disease (NIH) about Fighting Infection with Phages
(click here)

While bacteriophages have many uses, the use of bacteriophages have been approved for human consumption by Health Canada as an antimicrobial food processing aid ("Antimicrobial Food Processing Aid Uses on Red Meat and Poultry Meat for Which Health Canada has Expressed No Objection"[13]), as they have been demonstrated to reduce pathogens when applied to foods [14]. Bacteriophages have also been demonstrated to decrease pathogens in pet food (a bacteriophage preparation has been shown to decrease Salmonella in pet food up to 92% [15,16]). Safety of feeding bacteriophage-treated pet food has been demonstrated, as 12 dogs and cats were fed food with the phage additive with no changes in health outcomes [16]. As a result, bacteriophage application to pet foods has been proposed to decrease pathogens, which may decrease bacterial infection to pets and people through food and reduce food waste due to pet food recalls.

References

1. Heymann D. Control of Communicable Diseases Manual. Washington, DC; American Public Health Association; 2004.

2. Finley R, Reid-Smith R, Weese JS, Angulo FJ. Human Health Implications of Salmonella-Contaminated Natural Pet Treats and Raw Pet Food. *Clin Infect Dis*. 2006;42(5):686-691. doi:10.1086/500211

3. Cavallo, J S, Daly, R E, Seiferth J. Human Outbreak of Salmonella Typhimurium Associated with Exposure to Locally Made Chicken Jerky Pet Treats, New Hampshire, 2013. *Foodborne Pathog Dis*. 2015;12(5):441-446. doi:10.1016/j.physbeh.2017.03.040

4. Imanishi M, Rotstein, S D, Reimschuessel R, et al. Outbreak of Salmonella enterica serotype Infantis infection in humans linked to dry dog food in the United States and Canada, 2012. *J Am Vet Med Assoc*. 2014;244:545-553.

5. Center for Disease Control. Pet Food Safety. *US Dep Hum Heal Serv*. 2017:273956. https://www.cdc.gov/healthypets/resources/pet-food-tips_8x11_508.pdf.

6. Swanson KS, Carter RA, Yount TP, Aretz J, Buff PR. Nutritional Sustainability of Pet Foods. *Adv Nutr*. 2013;4(2):141-150. doi:10.3945/an.112.003335

7. Okin G. Environmental impacts of food consumption by dogs and cats. *Ecol Indic*. 2018;93:1043-1049. doi:10.1016/j.ecolind.2018.06.015

8. Su B, Martens P, Enders-Slegers MJ. A neglected predictor of environmental damage: The ecological paw print and carbon emissions of food consumption by companion dogs and cats in China. *J Clean Prod*. 2018;194:1-11. doi:10.1016/j.jclepro.2018.05.113

9. Helmer J. Food Print. Food Waste Is an Oft-Ignored Impact of Food Recalls. https://foodprint.org/blog/the-oft-ignored-environmental-impact-of-food-recalls-food-waste/. Published 2019. Accessed April 2020.

10. Sagman M. Dog Food Advisor. Dog Food Recalls. https://www.dogfoodadvisor.com/dog-food-recalls/. Published 2020. Accessed April 2020.

11. CDC.*Salmonella*. Outbreak of Multidrug-Resistant Salmonella Infections Linked to Contact with Pig Ear Pet Treats. https://www.cdc.gov/salmonella/pet-treats-07-19/index.html. Published 2019. Acessed April 2020.

12. Clokie MRJ, Millard AD, Letarov A V., Heaphy S. Phages in nature. *Bacteriophage*. 2011;1(1):31-45. doi:10.4161/bact.1.1.14942

13. Health Canada. Antimicrobial Food Processing Aid Uses on Red Meat and Poultry Meat for Which Health Canada has Expressed No Objection. 2019.

14. Wong CWY, Delaquis P, Goodridge L, Lévesque RC, Fong K, Wang S. Inactivation of Salmonella enterica on post-harvest cantaloupe and lettuce by a lytic bacteriophage cocktail. *Curr Res Food Sci*. 2020;2:25-32. doi:10.1016/j.crfs.2019.11.004

15. Heyse, Serena; Hanna, Leigh Farris; Woolston, Joelle; Sulakvelidze, Alexander; Charbonneau D. Bacteriophage Cocktail for Biocontrol of Salmonella in Dried Pet Food. *Virchows Arch*. 2015;455(4):97-103. doi:10.1002/9781444323870

16. Soffer N, Abuladze T, Woolston J, et al. Bacteriophages safely reduce Salmonella contamination in pet food and raw pet food ingredients . *Bacteriophage*. 2016;6(3):e1220347. doi:10.1080/21597081.2016.1220347

Consumer knowledge of and attitudes toward food safety, environmental sustainability and the use of bacteriophage applications in pet food

This questionnaire is authored by the University of British Columbia Animal Welfare Program and is not sponsored by a pet food company. **blue italics text is for notes, will not be included in survey*

<u>Part One</u>: To be answered **prior** to reading and viewing the accompanying educational information.

1. Do you have a dog(s), cat(s) or both? *Select all that apply.*

Dog:	1	2	3	4	5	more
Cat:	1	2	3	4	5	more

2. What do you feed your pet for meals? *Select all that apply.*
    a. Canned wet
    b. Dry kibble
    c. Commercial-prepared raw
    d. Home-prepared raw
    e. Leftovers
    f. Freeze dried or dehydrated
    g. Environmentally conscious protein source (e.g. insect protein) _______________
    **space for survey-takers to fill out text describing "other" option*
    h. Other __________________
    **space for survey-takers to fill out text describing "other" option*

3. What do you feed your pet for treats? *Select all that apply.*
    a. Canned wet
    b. Dry kibble
    c. Commercial-prepared raw
    d. Home-prepared raw
    e. Leftovers
    f. Freeze dried or dehydrated
    g. Snack product (e.g. 'Pup-Peroni' or 'Milk-bone')
    h. Environmentally conscious protein source (e.g. insect protein) _______________
    **space for survey-takers to fill out text describing "other" option*
    i. Other __________________
    **space for survey-takers to fill out text describing "other" option*

**answers provided for question 1-3 will be used to determine which questions each survey-taker will be directed to (for example, if someone does not indicate that they feed raw food, they will not be given any of the questions relating to raw food).*

4. Please rate the extent to which you agree/disagree with the following statements regarding **food safety** of pet food.

    a. It is important to me that the food I am feeding my pet is safe eat and will not make him/her sick.

    1 – Strongly disagree
    2 – Disagree
    3 – Neither agree or disagree
    4 – Agree
    5 – Strongly agree

    b. Bacteria can be found in raw pet foods and treats and can make pets and people sick

    1 – Strongly disagree
    2 – Disagree
    3 – Neither agree or disagree
    4 – Agree
    5 – Strongly agree

c. Bacteria can be found in canned pet foods and treats and can make pets and/or people sick

1 – Strongly disagree
2 – Disagree
3 – Neither agree or disagree
4 – Agree
5 – Strongly agree

d. Bacteria can be found in dry pet foods and treats and can make pets and/or people sick

1 – Strongly disagree
2 – Disagree
3 – Neither agree or disagree
4 – Agree
5 – Strongly agree

e. Bacteria can be found in freeze dried or dehydrated food and treats and can make pets and/or people sick

1 – Strongly disagree
2 – Disagree
3 – Neither agree or disagree
4 – Agree
5 – Strongly agree

5. Please rate how often you practice the following actions regarding **food safety** of pet food.

a. I wash my hands with soap and water after handling raw pet food or treats

1 – Never
2 – Rarely
3 – Sometimes
4 – Often
5 – Always

b. I wash my hands with soap and water after handling canned pet food or treats

1 – Never
2 – Rarely
3 – Sometimes
4 – Often
5 – Always

c. I wash my hands with soap and water after handling dry pet food or treats

1 – Never
2 – Rarely
3 – Sometimes
4 – Often
5 – Always

d. Where possible, I store pet food and/or treats away from where human food is stored or prepared

1 – Never
2 – Rarely
3 – Sometimes
4 – Often
5 – Always

e. I scoop pet food using my pet's food bowl

   1 – Never
   2 – Rarely
   3 – Sometimes
   4 – Often
   5 – Always

f. I use a clean, dedicated scoop/spoon/cup/utensil to serve pet food

   1 – Never
   2 – Rarely
   3 – Sometimes
   4 – Often
   5 – Always

g. I clean and disinfect surfaces that raw pet food touches

   1 – Never
   2 – Rarely
   3 – Sometimes
   4 – Often
   5 – Always

h. I thaw raw pet food on a countertop or in a sink

   1 – Never
   2 – Rarely
   3 – Sometimes
   4 – Often
   5 – Always

6. Please rate the extent to which you agree/disagree with the following statements regarding **sustainability** of pet food

   a. Organic pet food is more environmentally sustainable than conventional pet food

      1 – Strongly disagree
      2 – Disagree
      3 – Neither agree or disagree
      4 – Agree
      5 – Strongly agree

   b. A natural pet food option is more sustainable than a conventional one

      1 – Strongly disagree
      2 – Disagree
      3 – Neither agree or disagree
      4 – Agree
      5 – Strongly agree

   c. Sustainability is defined as the preservation of resources for future generations

      1 – Strongly disagree
      2 – Disagree
      3 – Neither agree or disagree
      4 – Agree
      5 – Strongly agree

d. Protein sources are variable in their environmental sustainability scoring (e.g. insect protein is generally a more sustainable protein option than beef)

1 – Strongly disagree
2 – Disagree
3 – Neither agree or disagree
4 – Agree
5 – Strongly agree

e. Food loss and waste is an inefficient use of resources and minimizing food waste promotes environmental sustainability

1 – Strongly disagree
2 – Disagree
3 – Neither agree or disagree
4 – Agree
5 – Strongly agree

f. Environmental sustainability of pet food is important to me

1 – Strongly disagree
2 – Disagree
3 – Neither agree or disagree
4 – Agree
5 – Strongly agree

g. Environmental sustainability guides my decisions when purchasing pet food

1 – Strongly disagree
2 – Disagree
3 – Neither agree or disagree
4 – Agree
5 – Strongly agree

7. Please rate the extent to which you agree/disagree with the following statements regarding **bacteriophages**.

a. A bacteriophage is a type of virus that infects and kills bacteria

1 – Strongly disagree
2 – Disagree
3 – Neither agree or disagree
4 – Agree
5 – Strongly agree

b. Bacteriophages are naturally occurring on earth

1 – Strongly disagree
2 – Disagree
3 – Neither agree or disagree
4 – Agree
5 – Strongly agree

c. Bacteriophages are exclusively produced in a laboratory

1 – Strongly disagree
2 – Disagree
3 – Neither agree or disagree
4 – Agree
5 – Strongly agree

d. Bacteriophages are considered one of the most abundant free-living entities on earth

1 – Strongly disagree
2 – Disagree
3 – Neither agree or disagree
4 – Agree
5 – Strongly agree

8. Please rate if you agree/disagree with the following statements regarding **bacteriophages** as antimicrobial agent applications to foods.

a. Bacteriophage applications are approved by Health Canada as food processing aids for human consumption

1 – Strongly disagree
2 – Disagree
3 – Neither agree or disagree
4 – Agree
5 – Strongly agree

b. Addition of bacteriophages to foods can decrease bacteria found on the food (e.g. salmonella)

1 – Strongly disagree
2 – Disagree
3 – Neither agree or disagree
4 – Agree
5 – Strongly agree

c. Bacteriophages are highly specific, and generally non-toxic to humans, animals and plants

1 – Strongly disagree
2 – Disagree
3 – Neither agree or disagree
4 – Agree
5 – Strongly agree

d. Bacteriophage applications to food are odourless and tasteless to humans

1 – Strongly disagree
2 – Disagree
3 – Neither agree or disagree
4 – Agree
5 – Strongly agree

9. Please rate the extent to which you agree/disagree with the following statements regarding **bacteriophages** in pet food.

a. I would feel comfortable eating food that has bacteriophage antimicrobial agents applied for food safety purposes

1 – Strongly disagree
2 – Disagree
3 – Neither agree or disagree
4 – Agree
5 – Strongly agree

b. I would feel comfortable feeding my pet food that has bacteriophage antimicrobial agents applied for food safety purposes

1 – Strongly disagree
2 – Disagree
3 – Neither agree or disagree
4 – Agree
5 – Strongly agree

c. I believe bacteriophage additives to food as antimicrobial agents would help environmental sustainability (due to decreased food waste as a result of recalls)

1 – Strongly disagree
2 – Disagree
3 – Neither agree or disagree
4 – Agree
5 – Strongly agree

<u>Part Two</u>: To be answered following reading and viewing the accompanying educational information.

10. Please indicate if you agree/disagree with the following statements regarding **bacteriophages**.

    a. A bacteriophage is a type of virus that infects and kills bacteria

       1 – Strongly disagree
       2 – Disagree
       3 – Neither agree or disagree
       4 – Agree
       5 – Strongly agree

    b. Bacteriophages are naturally occurring on earth

       1 – Strongly disagree
       2 – Disagree
       3 – Neither agree or disagree
       4 – Agree
       5 – Strongly agree

    c. Bacteriophages are exclusively produced in a laboratory

       1 – Strongly disagree
       2 – Disagree
       3 – Neither agree or disagree
       4 – Agree
       5 – Strongly agree

    d. Bacteriophages are considered one of the most abundant free-living entities on earth

       1 – Strongly disagree
       2 – Disagree
       3 – Neither agree or disagree
       4 – Agree
       5 – Strongly agree

11. Please rate if you agree/disagree with the following statements regarding **bacteriophages** as antimicrobial agent applications to foods.

    a. Bacteriophage applications are approved by Health Canada as food processing aids for human consumption

       1 – Strongly disagree
       2 – Disagree
       3 – Neither agree or disagree
       4 – Agree
       5 – Strongly agree

    b. Addition of bacteriophages to foods can decrease bacteria found on the food (e.g. salmonella)

       1 – Strongly disagree
       2 – Disagree
       3 – Neither agree or disagree
       4 – Agree
       5 – Strongly agree

    c. Bacteriophages are highly specific, and generally non-toxic to humans, animals and plants

       1 – Strongly disagree
       2 – Disagree
       3 – Neither agree or disagree
       4 – Agree

5 – Strongly agree

d. Bacteriophage applications to food are odourless and tasteless to humans

1 – Strongly disagree
2 – Disagree
3 – Neither agree or disagree
4 – Agree
5 – Strongly agree

12. Please rate the extent to which you agree/disagree with the following statements regarding **bacteriophage** additions to food as antimicrobial agents.

a. I would feel comfortable eating food that had bacteriophage additives

1 – Strongly disagree
2 – Disagree
3 – Neither agree or disagree
4 – Agree
5 – Strongly agree

b. I would feel comfortable feeding my pet food that had bacteriophage additives

1 – Strongly disagree
2 – Disagree
3 – Neither agree or disagree
4 – Agree
5 – Strongly agree

c. I believe bacteriophage additives to food would help environmental sustainability (due to decreased food waste as a result of recalls)

1 – Strongly disagree
2 – Disagree
3 – Neither agree or disagree
4 – Agree
5 – Strongly agree

13. What would prevent you from purchasing pet food that used bacteriophage applications as an antimicrobial agent?

a. It is unnatural

1 – Strongly disagree
2 – Disagree
3 – Neither agree or disagree
4 – Agree
5 – Strongly agree

b. I don't trust that it is safe

1 – Strongly disagree
2 – Disagree
3 – Neither agree or disagree
4 – Agree
5 – Strongly agree

c. I don't think it would help environmental sustainability

1 – Strongly disagree
2 – Disagree
3 – Neither agree or disagree

4 – Agree
5 – Strongly agree

d. I don't think it would help prevent food waste

1 – Strongly disagree
2 – Disagree
3 – Neither agree or disagree
4 – Agree
5 – Strongly agree

e. I don't think my pets would like the taste

1 – Strongly disagree
2 – Disagree
3 – Neither agree or disagree
4 – Agree
5 – Strongly agree

f. Other ____
**space for survey-takers to fill out text describing "other" option*

14. What is your gender
    a. Female
    b. Male
    c. Nonbinary
    d. Prefer to self-describe ________
    e. Prefer not to disclose

15. What is your age?
    a. 18-22 years
    b. 23-35 years
    c. 36-55 years
    d. 56-79 years
    e. >79 years

16. What is your level of education:
    a. Primary/Secondary
    b. High/Trade School
    c. University Education
    d. Postgraduate (Masters, PhD)

17. Are you in the medical field?
    a. Human Medical (MD, PA, RN, PhD)
    b. Animal medical (DVM, VMD, RVT)
    c. No

18. Would you consider participating in a trial investigating food preference of cats or dogs to pet food treated with bacteriophages as a microbial agent?
    a. Yes
    **contingent on selecting cat and saying yes, and answered that they have at least 1 cat in question 1, information below provided)*
    b. No

    The University of British Columbia Animal Welfare Program is conducting a study investigating the food preferences of cats to food with bacteriophages added as an antimicrobial agent. If you are interested in participating with your cat and would like to learn more, please contact *(Sasha or Bailey).*
